# Health-Related Quality of Life and Sense of Coherence among the Unemployed with Autotelic, Average, and Non-Autotelic Personalities: A Cross-Sectional Survey in Hiroshima, Japan

**DOI:** 10.1371/journal.pone.0073915

**Published:** 2013-09-12

**Authors:** Kazuki Hirao, Ryuji Kobayashi

**Affiliations:** Department of Occupational Therapy, School of Health Science and Social Welfare, Kibi International University, Takahashi, Okayama, Japan; Catholic University of Sacred Heart of Rome, Italy

## Abstract

**Background:**

Unemployment is known to have a negative effect on the quality of life (QOL) of individuals. However, the influence of an autotelic personality on QOL and SOC of unemployed individuals remains unclear. Our study compared health-related quality of life (HRQOL) and sense of coherence (SOC) among 3 groups: (i) an autotelic personality group (AP), which tends to “go with the flow,” (ii) an average group (AV), and (iii) a non-autotelic personality group (NAP).

**Methods:**

In October 2010, we conducted a cross-sectional survey among 140 job trainees not receiving unemployment benefits in Hiroshima, Japan. We collected 134 completed questionnaires. Autotelic personality was investigated using the Flow Experience Checklist, health-related quality of life was assessed using the Short Form (SF-8) Health Survey, and SOC was measured using the University of Tokyo Health Sociology version of the SOC3 scale (SOC3*–*UTHS).

**Results:**

The average age of participants was 36.14±11.54 year. Participants were classified into 3 groups based on daily activity values: 4+ for AP (*n* = 22), 1–3 for AV (*n* = 82), and 0 for NAP (*n* = 30). Significant differences were observed in mental component summary (MCS) score and SOC3–UTHS total scores in the ranking order of AP (highest), AV, and NAP.

**Conclusion:**

Our findings indicate a need to develop programs for facilitating AP among unemployed people to enhance mental QOL and SOC.

## Introduction

“Job loss is a life event in which paid employment is involuntarily taken away from an individual” [1]. There is strong evidence that becoming unemployed has a negative effect on mental health [2]–[4]. Therefore, it is important to maintain the quality of life (QOL) of the unemployed.

One of the core theories of positive psychology involves the concept of “flow” [5]. Flow has been defined as “the holistic sensation that people feel when they act with total involvement” [6]. We previously reported that flow experience has a positive effect on QOL of older people and college students [7]–[9].

Nakamura and Csikszentmihalyi defined the autotelic personality (AP) as spending more time in the flow state [10]. Ishimura et al. [11] reported that AP contributes toward having a good time and experiencing fewer negative emotions in Japanese college students. According to Asakawa, AP provides greater possibility of increased psychological well-being for people across all cultures [12]. These results suggest that AP has the potential to have a positive impact on QOL and sense of coherence (SOC; describing a concept that generates good health) [13], [14] of the unemployed. However, the influence of AP on QOL and SOC of unemployed persons remains unclear.

The aim of our study was to compare the health-related QOL (HRQOL) and SOC among 3 groups, those exhibiting AP, an average group (AV), and a non-autotelic personality group (NAP), among job trainees not receiving unemployment benefits.

## Methods

### Design

A cross-sectional survey was undertaken in October 2010. We compared HRQOL and SOC among the following 3 groups: (i) an AP group (AP), (ii) an AV group, and (iii) a NAP group.

### Participants

Participants included job trainees not receiving unemployment benefits from the Emergency training and employment support fund of the Ministry of Health, Labour and Welfare. Questionnaires were distributed to 140 job trainees at a career education center in Hiroshima, Japan. We collected 134 completed questionnaires, yielding a response rate of 95.7%.

The study followed the guidelines outlined in the Helsinki declaration. All participants were informed of the purpose and procedures of the study, written consent was obtained, and we explained how their anonymity would be preserved. Our study was approved by the ethics committee of Asahi Career Education Center. All data obtained were not connected to the subjects’ personal information when entered into Excel. After data input was completed, all original documents were destroyed by shredding.

### Measurements

Questionnaires were used to collect each participant’s basic information, including gender and age. Questionnaires also included the questions from the Flow Experience Checklist of Ishimura [15], Medical Outcome Study 8-Item Short-Form Health Survey (SF-8) [16], and the University of Tokyo Health Sociology version of the SOC3 scale (SOC3–UTHS) [17].

The Flow Experience Checklist is suitable for conveniently measuring flow in daily life. This checklist was used for extraction of the autotelic personality. Participants evaluated the elements of flow experience during 5 everyday life activities that they had described with reference to 10 items. There are also 6 items (2 for “challenge to goals” and 4 for “confidence in skills”) related to the “balance between challenges and skills,” which are a group of variables related to flow conditions and 4 items that measure “positive emotions and total involvement.” Participants were asked to evaluate experiences on a 7-point scale (1 = very strongly disagree to 7 = very strongly agree). The reliability and validity of this scale have been previously confirmed [15].

Health-related QOL was assessed using the Medical Outcome Study 8-Item Short-Form Health Survey (SF-8) [16]. This questionnaire is composed of 8 domains that measure health, with each item being answered on a 5- or 6-point scale. The 8 domains are physical functioning (PF), role-physical (RP), bodily pain (BP), general health perception (GH), vitality (VT), social functioning (SF), role-emotional (RE), and mental health (MH). In addition, there are 2 summary scores; the physical component summary (PCS) score and the mental component summary (MCS) score. The reliability and validity of this scale have been previously confirmed [16].

Sense of coherence (SOC) is a concept developed by Antonovsky that represents a personal ability to manage psychological stressors [14]. We used the SOC-3-UTHS, which has a 7-point scale (range, 3–21) [17]. SOC-3-UTHS consists of 3 items–manageability, meaningfulness, and comprehensibility. The reliability and validity of this scale have been previously confirmed [17].

### Statistical Analysis

Descriptive statistics were calculated for each variable. The χ^2^ test and one-way analysis of variance (ANOVA) were performed for the 3 groups (NAP, AV, AP) using the dependent variables of age, gender, SF-8 score, and SOC score. Tukey’s test was used for multiple comparisons. Variance homogeneity was examined using Levene’s test.

SPSS software (ver.19 for Windows) was used for all analyses. Results are represented as the mean±standard deviation (SD) and statistical significance was accepted at *P*<0.05.

## Results

### Extraction of Autotelic Personality

Each participant’s flow experience during important activities of daily life was assessed with reference to the report of Csikszentmihalyi et al. [18]. Because flow is experienced when the challenge to achieve a goal is balanced by sufficiently high skills, the challenge to achieve goals (challenge level: M = 7.67, SD = 3.88) and confidence in skills (skill level: M = 16.81, SD = 5.86) were employed for the assessment. Based on the mean values of these items, the participants were classified into a flow group based on the levels of challenge and skills. AP was identified on the basis of the number of activities that indicated flow experience [10]. Based on the mean values (M = 1.90) with a margin of ±1 SD (SD = 1.57) of the number of flow activities, 3 groups related to AP characteristics were classified. For the NAP group (*n* = 30, 22.4%), the number of flow activities was 0. For the average group (*n* = 82,61.2%), the number of flow activities was 1–3. For the AP group (*n* = 22, 16.4%), the number of flow activities was more than 4.

### Demographic Characteristics

Application of Levene’s test yielded results that showed homoscedasticity. The demographic characteristics of participants in our study are shown in Table 1. The results of χ^2^ test and one-way ANOVA did not show significant differences among the 3 groups with respect to gender (χ^2^ = 1.94, *P = *0.38) or age [F(2,131) = 1.33, *P = *0.27]. The average age of the participants was 36.14±11.54 year.

**Table 1:** Demographic characteristics of the three groups (compared using the χ^2^ test and one-way ANOVA).

	NAP (*n* = 30)	AV (*n* = 82)	AP (*n* = 22)		
Gender				χ^2^/F	*P* value
Male	6 (20)	15 (18.3)	7 (31.8)	1.94	0.38
Female	24 (80)	67 (81.7)	15 (68.2)		
Age (year)	33.17±12.13	36.72±10.66	37.91±13.59	1.33	0.27

NAP = non-autotelic personality group; AVG = average group; AP = autotelic personality group.

Data are shown as the number (%) or mean±SD.

### Health-related QOL (SF-8 Scores)

Application of Levene’s test yielded results that showed homoscedasticity. The SF-8 scores of the 3 groups are shown in Table 2. One-way ANOVA showed significant differences among the 3 groups with respect to GH [F (2,131) = 7.98, *P = *0.001], VT [F (2,131) = 8.85, *P = *0.000], SF [(F (2,131) = 3.43, *P = *0.035], RE [F (2,131) = 4.56, *P = *0.012], MH [F (2,131) = 5.41, *P = *0.006], and MCS [F (2,131) = 8.04, *P = *0.001]. Multiple comparison analysis using Tukey’s test showed GH in the AP group was significantly higher than that in the NAP group (52.56±6.24 vs. 44.42±8.71, *P* = 0.000); GH in the AV group was significantly higher than that in NAP group (48.67±7.09 vs. 44.42±8.71, *P = *0.021). VT in the AP group was significantly higher than that in the AV group (53.36±6.17 vs. 49.25±6.34, *P* = 0.029); VT in the AP group was significantly higher than that in the NAP group (53.36±6.17 vs. 45.56±7.65, *P* = 0.000); and VT in the AV group was significantly higher than that in NAP group (49.25±6.34 vs. 45.56±7.65, *P* = 0.027). In addition, SF in the AP group was significantly higher than that in the NAP group (50.23±5.84 vs. 44.26±9.91, *P* = 0.028); moreover, there was a significant difference in MH (48.44±5.52 vs. 41.85±7.54, *P* = 0.004). RE in the AP group was significantly higher than that in the AV group (50.74±5.32 vs. 46.67±6.94, *P* = 0.032). RE in the AP group was significantly higher than that in the NAP group (50.74±5.32 vs. 45.27±6.78, *P* = 0.011). Moreover, MCS in the AP group was significantly higher than that in the AV group (49.48±5.14 vs. 45.44±6.46, *P* = 0.037). MCS in the AP group was significantly higher than that in the NAP group (49.48±5.14 vs. 41.88±8.43, *P* = 0.000), and MCS in the AV group was significantly higher than that in the NAP group (45.44±6.46 vs. 41.88±8.43, *P* = 0.040).

**Table 2:** SF-8 scores of the three groups (representing health-related QOL).

	NAP (*n* = 30)	AV (*n* = 82)	AP (*n* = 22)		
	Mean±SD	Mean±SD	Mean±SD	F	Multiple comparison
Physical functioning	47.2±8.4	48.65±8.28	49.88±5.09	0.76	n.s.
Role-physical	47.1±5.83	48.98±6.40	50.88±4.14	2.57	n.s.
Bodily pain	47.26±10.29	46.26±8.56	48.24±9.43	0.46	n.s.
General health perception	44.42±8.71	48.67±7.09	52.56±6.24	7.98**	NAP<AV, AP
Vitality	45.56±7.65	49.25±6.34	53.36±6.17	8.85**	NAP<AV<AP
Social functioning	44.26±9.91	47.28±8.04	50.23±5.84	3.43*	NAP<AP
Role-emotional	45.27±6.78	46.67±6.94	50.74±5.32	4.56*	NAP, AVG<AP
Mental health	41.85±7.54	45.40±7.55	48.44±5.52	5.41**	NAP<AP
PCS	47.3±8.44	48.21±6.30	49.73±6.6	0.8	n.s.
MCS	41.88±8.43	45.44±6.46	49.48±5.14	8.04**	NAP<AVG<AP

Data are shown as the mean±SD.

* *P*<0.05;

** *P*<0.01.

Health-related QOL: health-related quality of life; SF-8: Medical Outcomes Study, 8-Item Short-Form Health Survey; PCS: physical component summary; MCS: mental component summary; NAP = non-autotelic personality group; AV = average group; AP = autotelic personality group.

### SOC3 Scores

Application of Levene’s test yielded results that showed homoscedasticity. The SOC3 scores in the 3 groups are shown in Table 3. One-way ANOVA showed significant differences among the 3 groups with respect to SOC total score [F (2,131) = 18.41, *P* = 0.000], manageability [F (2,131) = 12.41, *P* = 0.000], meaningfulness [F(2,131) = 16.19, *P = *0.000), and comprehensibility [F(2,131) = 12.38, *P* = 0.000]. Multiple comparison analysis using Tukey’s test showed that the total SOC scores in the AP group were significantly higher than that in the AV group (17±3.15 vs. 14.74±3.16, *P* = 0.02); SOC in the AP group was significantly higher than that in the NAP group (17±3.15 vs. 11.33±4.35, *P* = 0.000), and SOC in the AV group was significantly higher than that in NAP group (14.74±3.16 vs. 11.33±4.35, *P = *0.000). Manageability in the AP group was significantly higher than that in NAP group (5.32±1.43 vs. 3.6±1.5, *P* = 0.000), and significantly higher in the AV group than in the NAP group (4.77±1.23 vs. 3.6±1.5, *P = *0.000). In addition, meaningfulness in the AP group was significantly higher than that in the NAP group (6.18±0.96 vs. 4.2±1.63, *P* = 0.000) and was significantly higher in the AV group than in the NAP group (5.51±1.29 vs. 4.2±1.63, *P* = 0.000). Comprehensibility was significantly higher in the AP group than in the AV group (5.5±1.34 vs. 4.46±1.34, *P* = 0.008), in the AV group than the NAP group (5.5±1.34 vs. 3.53±1.63, *P* = 0.000), and in the AP group than that in the NAP group (4.46±1.34 vs. 3.53±1.63, *P* = 0.007).

**Table 3:** SOC3 scores of the three groups.

	NAP (*n* = 30)	AV (*n* = 82)	AP (*n* = 22)		
	Mean±SD	Mean±SD	Mean±SD	F	Multiple comparison
SOC total scores	11.33±4.35	14.74±3.16	17±3.15	18.41**	NAP<AV<AP
Manageability	3.6±1.5	4.77±1.23	5.32±1.43	12.41**	NAP<AV, AP
Meaningfulness	4.2±1.63	5.51±1.29	6.18±0.96	16.19**	NAP<AV, AP
Comprehensibility	3.53±1.63	4.46±1.34	5.5±1.34	12.38**	NAP<AV<AP

Data are shown as the mean±SD.

* *P*<0.05;

** *P*<0.01.

SOC: sense of coherence; SOC-3: University of Tokyo Health Sociology version of the SOC scale; NAP = non-autotelic personality group; AV = average group; AP = autotelic personality group.

## Discussion

In our study, the proportion of participants showing AP (*n* = 22) was 16.4%. In previous studies, approximately 20% (16.7% of Japanese, 16% of Americans, 23% of Germans) reported high degree of flow experience [10], [11]. These studies had similar findings to our study. Racial differences may be less in AP.

The results of our study showed that AP was associated with a significantly higher mental QOL than AV and NAP in unemployed people. Previous studies reported that flow was positively related to aspects of psychological well-being in Japanese college students [19]. Psychological well-being was considerably high during periods of flow in Japanese college students [12]. Japanese college students with AP reported more feelings of fulfillment and greater satisfaction with their lives [20]. Thus, our results were similar to the results of the this study. These results suggested that AP has a positive impact on mental health in different environments and age groups. We believe that AP has the potential to improve the mental QOL of unemployed people. To that end, there is a need for intervention to promote the flow in everyday life.

The results of our study showed that AP was associated with a significantly higher SOC than AV and NAP in unemployed people. SOC is proposed to have a buffering effect on stressors [21]. Those with AP tend to enjoy daily life of their own accordance and have a high SOC, and stress is uncommon among them. In our previous study, we elucidated that increased flow reduces subjective report of stress [8].

Jonathan Lutz pointed out that SOC and concept of flow are extremely similar–these two constructs perhaps may be regarded as single dynamic [22]. In this way, the concepts of flow and SOC may be extremely close.

Individuals with AP use active coping strategies in daily life more often than those with less AP among Japanese college students [20]. Furthermore, those with AP reported active commitments to college life and searching for future careers [20]. In this way, unemployed individuals with AP may be active against reinstatement, there is a possibility to spare no effort for it. In addition, because SOC of unemployed people was significantly lower than that of the employer [23], we believe it is necessary to increase the SOC by accelerating the flow in everyday life.

The limitation of our study is that it is a cross-sectional study, which attributions about the direction of causality between variables. Accordingly, a longitudinal study should be performed in the future.

## Conclusion

The findings suggest that AP was associated with a significantly higher mental QOL and SOC than AV and NAP among unemployed people. We need to consider developing programs that may facilitate the flow experience to enhance mental QOL and SOC among unemployed people.
